# Supplementary material for: Investigating the running abilities of Tyrannosaurus rex using stress-constrained multibody dynamic analysis
Source: PeerJ. 2017 Jul 18;5:e3420. doi: 10.7717/peerj.3420 (PMC5518979; doi:10.7717/peerj.3420)
Supplement: Supplemental Information 1 [file peerj-05-3420-s001.docx]

**Multiphysics modelling limits *Tyrannosaurus rex* to walking speeds**

Sellers WI, Pond SB, Brassey CA, Manning PL, Bates KT.

**Supplementary Information**

**Body Mass Calculation**

The body mass of BHI 3033 was calculated using the minimum convex hull methodology (1) using an extended dataset combined from our previously published work on mammal and bird body masses (1-4). This data is shown in Table S1 and includes our full species list but excludes the birds where we only had an eviscerated mass. The predictive equation for body mass was generated from this data and using a linear fit with the data constrained to pass through the origin since as we have argued previously (1), we believe this represents the simplest biological model. Lines of best fit were calculated using ordinary least squares (OLS), major axis (MA), and standardized major axis (SMA) approaches using the smatr library for R (5). The best fit slopes were: OLS 1067.352 ±37.03; SMA 1073.292 ±37.03; MA 1079.266 ±37.49 and the SMA value was used to calculate the mass for BHI 3033 which had a minimum convex hull volume of 6.714 m^3^ and therefore a body mass of 7206.496 kg ±248.66. The relationship between minimum convex hull volume and body mass is illustrated in Figure 1S which also shows the SMA relationship and the predicted value for BHI 3033.

| Species | Minimum Convex Hull Volume (m^3^) | Body Mass (kg) |
| --- | --- | --- |
| *Struthio camelus* | 0.0717 | 60.7 |
| *Casuarius casuarius* | 0.0172 | 27 |
| *Dromaius novaehollandiae* | 0.0214 | 20.06 |
| *Rhea americana* | 0.0177 | 16.3 |
| *Rhea pennata* | 0.0159 | 14.9 |
| *Apteryx australis* | 0.0011 | 2.96 |
| *Apteryx australis lawryi* | 0.0014 | 2.41 |
| *Branta leucopsis* | 0.0011 | 1.69 |
| *Numida meleagris* | 0.001 | 1.4 |
| *Bison bison* | 0.4733 | 558.5 |
| *Bos taurus* | 0.2191 | 323.7 |
| *Camelus dromedaries* | 0.3211 | 427 |
| *Cervus elaphus* | 0.084 | 89.5 |
| *Dicerorhinus sumatrensis* | 0.3614 | 470.3 |
| *Elephas maximus* | 2.093 | 2352 |
| *Equus caballus* | 0.3698 | 517.5 |
| *Giraffa camelopardalis* | 0.4349 | 638.2 |
| *Loxodonta africana* | 2.748 | 2734.9 |
| *Megaloceros giganteus* | 0.3012 | 435.6 |
| *Rangifer tarandus* | 0.0757 | 95.8 |
| *Sus scrofa* | 0.0779 | 107.4 |
| *Tapirus indicus* | 0.1699 | 295.3 |
| *Ursus maritimus* | 0.1109 | 206.1 |
| *Chlorocebus aethiops* | 0.0037 | 3.78 |
| *Macaca fuscata* | 0.0051 | 6.6 |
| *Saimiri sciureus* | 0.0006 | 0.759 |
| *Hylobates agilis* | 0.0054 | 6.75 |
| *Hylobates lar* | 0.0066 | 8.57 |
| *Gorilla gorilla* | 0.0957 | 176 |
| *Pan troglodytes* | 0.0418 | 52.9 |
| *Pongo pygmaeus* | 0.0325 | 45 |
| *Homo sapiens* | 0.0491 | 68.9 |
| *Streptopelia decaocto* | 2.04E-04 | 0.201 |
| *Columba livia* | 1.16E-04 | 0.29 |
| *Columba palumbus* | 3.38E-04 | 0.305 |
| *Phaps chalcoptera* | 2.14E-04 | 0.249 |
| *Ducula aenea* | 3.48E-04 | 0.483 |
| *Columba guinea* | 1.05E-04 | 0.158 |
| Ocyphaps lophotes | 6.75E-05 | 0.107 |

Table S1. This table lists the convex hull volumes and body masses used to derive the scaling equation used to predict BHI 3033. Various approaches are used to generate both the minimum convex hull volume and the body mass and these are detailed in the original papers (1-4).

Figure S1. These plots show the relationship between minimum convex hull volume and body mass for the data in table S1 using a SMA linear fit through the origin. The red lines show the 95% confidence limits for the line of best fit, and the predicted value for BHI 3033 is also shown on the lower plot.

**References**

1. Sellers WI, Hepworth-Bell J, Falkingham PL, Bates KT, Brassey CA, Egerton VM, et al. Minimum convex hull mass estimations of complete mounted skeletons. Biology Letters. 2012;8:842-5.

2. Brassey CA, Holdaway RN, Packham AG, Anné J, Manning PL, Sellers WI. More than one way of being a moa: differences in leg bone robustness map divergent evolutionary trajectories in Dinornithidae and Emeidae (Dinornithiformes). PLoS ONE. 2013;8(12):e82668.

3. Brassey CA, Sellers WI. Scaling of Convex Hull Volume to Body Mass in Modern Primates, Non-Primate Mammals and Birds. PLoS ONE. 2014;9(3):e91691.

4. Brassey CA, O’Mahoney TG, Kitchener AC, Manning PL, Sellers WI. Convex-hull mass estimates of the dodo *(Raphus cucullatus*): application of a CT-based mass estimation technique. PeerJ. 2016;4(1):e1432.

5. Warton DI, Duursma RA, Falster DS, Taskinen S. smatr 3 - an R package for estimation and inference about allometric lines. Methods in Ecology and Evolution. 2012;32(2):257-9.
